# Continuous Ketamine Infusion as a Treatment for Refractory Facial Pain

**DOI:** 10.7759/cureus.35638

**Published:** 2023-03-01

**Authors:** Roxana Garcia, QiLiang Chen, Edmund Posadas, Johnathan Tran, Albert Kwon, Xiang Qian

**Affiliations:** 1 Department of Orthopedic Surgery, Physical Medicine, and Rehabilitation, Stanford Health Care, Palo Alto, USA; 2 Department of Anesthesiology, Stanford Health Care, Palo Alto, USA; 3 Department of Anesthesiology, University of California San Diego, La Jolla, USA; 4 Department of Anesthesiology, The Permanente Medical Group, Oakland, USA

**Keywords:** safety and efficacy, chronic pain management, ketamine infusion, atypical facial, trigeminal neuralgia

## Abstract

Complex orofacial pain disorders, such as trigeminal neuralgia (TN) and atypical facial pain (AFP), can be excruciating and debilitating during attacks. Ketamine, an N-methyl-D-aspartate (NMDA) antagonist, is a powerful analgesic that has been used to treat various chronic pain conditions, but its role in treating complex facial pain has only been recently explored. In this retrospective case series, we reviewed the efficacy of continuous ketamine infusion for 12 patients with facial pain refractory to medical treatment. Patients who presented with a diagnosis of TN were more likely to have significant and sustained pain relief after receiving ketamine infusion. By contrast, those who did not respond to the treatment were more likely to have a diagnosis of AFP. The current report suggests a fundamental difference between these two facial pain disorders in their respective underlying pathophysiology and supports the use of continuous ketamine infusion for refractory TN, but not AFP.

## Introduction

Orofacial pain is a class of complex and debilitating pain conditions that are challenging to diagnose and treat [1]. The International Classification of Orofacial Pain (ICOP) committee most recently introduced classifications for clinical orofacial pain conditions based on the International Classification of Headache Disorders (ICHD-3) as an attempt to better diagnose and identify targeted treatments for each disease variation [2]. Based primarily on the presentation and history of the pain, the ICOP divides orofacial pain into six main groups, including trigeminal neuralgia (TN) and idiopathic facial pain syndromes [1].

TN, one of the most common forms of severe orofacial pain, is characterized by recurrent severe paroxysmal stabbing or electric shock-like pain in the trigeminal nerve distribution [3,4]. The cause of TN is likely multifactorial. However, the most accepted pathophysiology involves trigeminal axonal demyelination and neuronal hyperexcitability secondary to either neurovascular compression or other primary demyelination diseases [3]. Anticonvulsants, such as carbamazepine and oxcarbazepine, are considered the first-line pharmacological treatment for TN [3]. More aggressive interventions, such as neurovascular decompression and radiofrequency ablation, are reserved for patients who fail pharmacological therapy.

On the other hand, atypical facial pain (AFP), also known as persistent idiopathic facial pain, is a diagnosis of exclusion. It is characterized by persistent facial pain that is beyond the trigeminal nerve distribution and is often associated with neuropathic pain quality, such as dull, aching, or nagging [2]. Unfortunately, its pathophysiology is poorly understood [5]. Unlike TN, AFP is often associated with a traumatic event, such as major dental surgeries [3,5,6]. Although most studies on the mechanism of AFP are associative and inconclusive, the pharmacological treatment for this disease suggests that there are some degrees of central and peripheral neuronal hyperexcitability [7]. First-line treatment commonly involves anticonvulsants, such as gabapentin and pregabalin, as well as antidepressants, such as amitriptyline and duloxetine [1].

However, options for medical management remain limited when TN or AFP patients become refractory to their respective first-line treatments. Ketamine infusion has shown promise in treating neuropathic pain. Being an analgesic and sedative agent that acts primarily as a non-competitive antagonist of N-methyl-D-aspartate (NMDA) receptors and with endogenous opioid receptors at high thresholds, ketamine is thought to limit central sensitization through plasticity and the descending antinociceptive pathway [8-11]. Continuous infusion may impact central sensitization more than intermittent infusion [9]. Despite its efficacy as a general analgesic, the literature on ketamine infusions for refractory facial pain is limited. Intramuscular or parenteral infusion of ketamine has been associated with neuropathic pain relief in chronic orofacial pain, migraines, acute pain after oral surgery, chronic regional pain syndrome (CRPS), and refractory TN [8,12-15]. This current retrospective case series aimed to explore the clinical efficacy of continuous ketamine infusions in patients with refractory orofacial pain, particularly those with AFP or TN, and identify key clinical characteristics that were associated with treatment responders and non-responders.

## Materials and methods

This study was performed in accordance with relevant guidelines and regulations. The research protocol was approved by the Institutional Review Board Committee at Stanford University (protocol #52966). A total of 280 patients were admitted to the inpatient chronic pain service at the Stanford Medical Center for continuous ketamine infusion from 2011 to 2018. Inpatient ketamine infusion is a part of the standard treatment protocol at Stanford University Hospital and is regularly offered to patients with resistant facial pain who have failed pharmacological management. Of the 280 patients who received ketamine infusion, 12 patients were identified for further review with the specific hospital admission diagnosis of “refractory facial pain,” “atypical facial pain,” or “trigeminal neuralgia.” These 12 patients formed the basis of this retrospective pilot study. These patients’ records were also associated with descriptions of “facial pain,” “oral pain,” “facial CRPS,” or “trigeminal neuropathy.” Given the classification of “persistent idiopathic facial pain” did not exist before 2020, this search term was omitted. Patients’ electronic medical records were reviewed for demographic data, medical history, psychiatric history, body mass index (BMI), medications prescribed prior to admission, prior procedural interventions, and medications received during admission.

All 12 of these patients were admitted for five to seven days of continuous ketamine infusion according to the institutional protocol, which started ketamine infusion (non-weight-based) at 10 mg/h and titrated up to 50 mg/h based on the treatment efficacy and patient tolerance. Pain scores were recorded every four hours, and vitals were recorded every six hours. Average daily pain scores throughout the admission were calculated for each patient. Based on the methods recommended in the Initiative on Methods, Measurement, and Pain Assessment in Clinical Trials (IMMPACT) guidelines, “responders” were defined as patients with a significant reduction (50% or greater) in the pain score or a pain score of 0 at the time of discharge and sustained pain relief for more than two weeks [16]. The remaining patients were considered “non-responders.” Similar benchmarks have been used in previously published studies to assess the impact of ketamine infusion in chronic pain patients [16-18]. Furthermore, >50% reduction in pain intensity score aligns with published guidelines for chronic pain studies [16-18]. Patients were followed up for six months after ketamine infusion.

Adverse effects during hospital admission were documented and included psychomimetic symptoms (dysphoria, hallucinations), somnolence, pre-syncope, nausea, vomiting, and elevated blood pressure. Statistical analyses were performed using GraphPad Prism software, version 9. Quantitative data were presented as mean ± standard deviation. Qualitative data were expressed as frequency and percentage. Student’s t-test of significance for independent samples was used for comparing two means. Patient characteristics (including patients’ age, length of pain history, BMI, and length of infusion) between the responder group and the non-responder group were compared. Comparisons between patients’ pain scores were done with the Mann-Whitney test. Chi-square tests were performed for comparisons between categorical data (e.g., the number of patients with depression and/or anxiety). A p-value less than 0.05 was considered statistically significant.

## Results

Patient demographic data are summarized in Table 1. There was no significant difference in age, chronic pain history, sex, BMI, psychiatric history, pre-admission opioid use, pre-admission anticonvulsant use, and pre-treatment pain score between the responder and non-responder groups (Table 1).

**Table 1:** Patient demographics This table gives a summary of patient demographics of the ketamine infusion responders and non-responders. There was a significantly greater proportion of trigeminal neuralgia patients in the responder group than in the non-responder group. Otherwise, no significant difference in patient characteristics was found between the two groups. AFP: Atypical facial pain. ^†^ Student’s t-test; ^£ ^Chi-square; ^‡^ Mann-Whitney; * Statistically significant.

	Responders (n = 7)	Non-responders (n = 5)	p-value
Age (years ± SD)	49.9 ± 12.7	62.8 ± 5.9	^†^0.062
Chronic pain history (years ± SD)	8.3 ± 5.9	14.6 ± 6.5	^†^0.110
Sex (Male:Female)	1:6	1:4	^£^0.793
BMI	27.2 ± 7.1	24.0 ± 4.0	^†^0.26
Patients with depression and/or anxiety (n)	5	4	^£^0.735
Pre-admission opioids (n)	5	3	^£^0.679
Pre-admission anticonvulsants (n)	7	4	^£^0.217
Pre-treatment pain score (average ± SD)	7.3 ± 2.6	8.2 ± 1.5	^‡^0.679
Diagnosis (n)	TN: 6; AFP: 1	TN: 1; AFP: 4	^£^0.023*

Differences in the treatment protocol and patient characteristics between the responder and non-responder groups were investigated. Interestingly, there were no significant differences in infusion duration or maximum infusion rate between responders and non-responders. However, the weight-corrected infusion rate showed that the responders required a lower dose of ketamine to achieve adequate pain relief (0.37 ± 0.12 vs. 0.54 ± 0.90 mg/kg/h, p = 0.034) as shown in Table 2. However, when the admission diagnoses were analyzed, it was noted that patients with TN were more likely to respond to ketamine infusion compared to patients with AFP (Chi-square = 5.18, df = 1, p = 0.023; odd ratio = 24, 95% CI = 1.14-505.22, p = 0.041) as shown in Table 2. Furthermore, the responder group appeared to have a younger average age compared to the non-responder group, although this was not statistically significant (49.9 ± 12.7 vs. 62.8 ± 5.9, p = 0.062) as shown in Table 1.

**Table 2:** Treatment responses This table gives a summary of the treatment responses to continuous ketamine infusion for five to seven days. There was a significant reduction in the pain score and persistent relief in the responder group compared to the non-responder group. ^AFP: Atypical facial pain.^ ^† ^Student’s t-test; ^£ ^Chi-square: ^‡ ^Mann-Whitney; * Statistically significant.

	Responders (n = 7)	Non-responders (n = 5)	p-value
Changes in pain score post-ketamine treatment (average ± SD)	-6.4 ± 2.3	-2.4 ± 2.6	^‡^0.018*
Duration of ketamine infusion (days ± SD)	5.7 ± 0.5	5.8 ± 0.8	^†^0.57
Maximum infusion rate (mg/hr ± SD)	27.1 ± 3.9	34.0 ± 9.6	^†^0.12
Maximum infusion rate (mg/kg/hr ± SD)	0.37 ± 0.12	0.54 ± 0.9	^†^0.034*
Duration of post-ketamine pain relief (weeks ± SD)	10.3 ± 9.9	0 ± 0	^†^0.04*
Diagnosis (n)	TN: 6; AFP: 1	TN: 1; AFP: 4	^£^0.023*

To better characterize the degree of pain relief from continuous ketamine infusion, the change in pain scores of the responder group was compared with previously published data [13]. Adopting the historic data from the study of Mogahed et al. on the efficacy of intermittent ketamine infusion for TN patients (e.g., two hours of infusion at a dose of 0.4 mg/kg every four days for three sessions), patients in our responder group, who were predominantly TN patients, had a greater reduction in pain scores compared to the patients who received intermittent ketamine infusion (n = 7, -6.4 ± 2.3 vs n = 50, -3.2 ± 0.6, respectively, df = 36, p < 0.001). All patients reported a decrease in pain scores, although this was only statistically significant in the responder group. The responder group was also more likely to have a sustained pain reduction effect after ketamine infusion, with an average duration of 10.3 weeks of pain relief (Table 2), and for two cases, it lasted up to six months.

The side effects of ketamine infusion were minimal and temporary. Among the 12 patients during the admission period, two patients reported pre-syncopal symptoms such as lightheadedness and dizziness, one experienced somnolence, one experienced hallucination, one had nausea, and one had elevated blood pressure. These symptoms were self-limiting within hours once infusion rates were adjusted to a lower dose or stopped completely. The as-needed medications used by the patients included clonidine and lorazepam. There were no complaints of long-term sequelae on follow-up appointments.

## Discussion

Orofacial pain is challenging to treat, in part, because of a lack of pathophysiological understanding of the subtypes of orofacial pain [5]. In the current study, a five- to seven-day course of continuous ketamine infusion was able to produce significant lasting pain relief in some patients with refractory facial pain, particularly in those with TN. Responders reported up to 10 weeks (2½ months) of significant pain relief (e.g., >50% of pain reduction); the majority of responders were patients with TN. By contrast, non-responders did report pain relief, but this was neither significant (e.g., <50% pain reduction) nor lasting. Patients with AFP were less likely to gain pain relief from ketamine despite a higher infusion dose. This difference in response may support differences in pathophysiology between these facial pain disorders [3,4].

There is limited research to guide the use of continuous or intermittent ketamine for chronic neuropathic or nonneuropathic chronic pain [19]. Inpatient monitoring during continuous ketamine infusion in the current study allowed the safe delivery of a higher dose of ketamine compared to intermittent ketamine infusion [13]. There are known biological mechanisms consistent with the notion that continuous ketamine infusion may lead to better analgesia in the perioperative period and may be more suited than intermittent boluses in treating conditions related to central sensitization [9,20]. Although continuous ketamine infusion is likely more costly than intermittent infusion, a greater analgesic effect may justify the investment of resources. However, this study is limited by its small sample size. Future randomized controlled studies specifically designed for investigating the pharmacological and therapeutic differences between continuous and intermittent ketamine infusion for orofacial pain will be required.

Although failed to reach statistical significance, our current data demonstrated a trend that younger participants respond more favorably to ketamine infusion than older participants. There are no published studies that examine age as an independent factor in ketamine infusion for complex orofacial pain disorders. In the studies of serial ketamine infusions for treatment-resistant depression, younger age may be associated with a faster response but may not be associated with durability or efficacy [21]. In ketamine infusion for acute sickle cell crises, younger age may predict a greater reduction in pain scores [18]. Future studies with a bigger sample size can further delineate this potential relationship.

One of the advantages of an inpatient continuous ketamine infusion is that it allows trained staff to titrate ketamine to the desired pain-relieving effect for each patient while monitoring for adverse events from ketamine administration. This allows for close inpatient monitoring of any psychiatric symptoms. The incidence of ketamine addiction is unknown due to the lack of large-scale studies, but nevertheless, ketamine's potential for addiction should be considered when patients are treated with intermittent IV infusions [22,23]. In this study, the infusion dose was well below the anesthetic or recreational dosage. Additionally, patients were closely followed post-infusion for both pain relief and dependence tendency [24]. None of the patients in the current study displayed addictive behaviors. Furthermore, although there is no standard definition of what dose of ketamine is considered “subanesthetic,” anesthetic doses of ketamine are generally 1.5-2 mg/kg or higher and given as a bolus [19]. In our protocol, subanesthetic doses of ketamine were used, and thus serious side effects, such as hemodynamic changes, were not seen. Adverse effects commonly associated with subanesthetic doses of ketamine include psychomimetic symptoms (dysphoria, hallucinations, nightmares, and vivid dreams), blurry vision or diplopia, and nausea or vomiting [19]. Repeated ketamine exposure may increase sensitivity to cognitive side effects [25]. As seen in the current study, close monitoring spares patients from higher than required ketamine doses and limits long-term sequelae. Furthermore, with the complex pharmacokinetics of ketamine on NMDA receptors and endogenous opioid receptors, careful titration of dosage would be required in chronic patients with multiple comorbidities and polypharmacy [26].

## Conclusions

Overall, our study suggests that continuous ketamine infusion provides a greater and longer pain relief for patients with refractory TN than for those with AFP. However, there are several major limitations, given the retrospective and case-series nature of the study. First, the long-term effects of five to seven days of continuous ketamine infusion remain unknown, given the small sample size and the short-term follow-up period. The lack of a control arm for the responder group could further confound the interpretation of the data. Finally, given the clinical sources required for inpatient infusion treatment, the missing data on treatment cost can limit its application in settings with scarce resources. Research on technical parameters of ketamine infusion, including dosing, length, and frequency of infusion, will also be required. Future randomized controlled studies with larger sample sizes and longer follow-up periods would be needed to further delineate the effects of continuous ketamine infusion as a therapy for refractory facial pain.
